# Research Progress of Drug Prophylaxis for Lens Capsule Opacification after Cataract Surgery

**DOI:** 10.1155/2020/2181685

**Published:** 2020-07-04

**Authors:** Rong-Pei Zhang, Zheng-Gao Xie

**Affiliations:** ^1^Department of Ophthalmology, Northern Jiangsu People's Hospital Affiliated to Yangzhou University, Yangzhou 225001, China; ^2^Graduate School of Dalian Medical University, Dalian Medical University, Dalian 116044, China; ^3^Department of Ophthalmology, Nanjing Drum Tower Hospital, The Affiliated Hospital of Nanjing University Medical School, Nanjing 210008, China

## Abstract

Phacoemulsification combined with intraocular lens (IOL) implantation is the international standard operation procedure for cataract and has been generalized worldwide. However, lens capsule opacification, one of the common complications after cataract surgery, impacts the recovery of patients' visual function to a large extent. Lens capsule opacification has two types, anterior capsule opacification (ACO) and posterior capsule opacification (PCO), according to the location. There is not an accepted approach to treat ACO. Nd : YAG laser capsulotomy, the common treatment of PCO, can effectively improve the vision, but may cause a series of complications and is inappropriate for children who are too young to cooperate with this treatment. It is generally known that the responses of lens epithelial cells (LECs) after cataract surgery, including cell proliferation, migration, and epithelial-mesenchymal transition (EMT), play a key role in the pathogenesis of lens capsule opacification. Scholars found that substantial drugs can reduce the occurrence of lens capsule opacification by inhibiting, clearing, or killing LECs, and made great efforts as well as innovations on the exploration of drug species or modes of administration. This article is a systematic interpretation and elaboration about how to prevent lens capsule opacification after cataract surgery via different drugs.

## 1. Introduction

Cataract is the principal cause of blindness worldwide, leading to at least 53 million people blind up to now [1]. Phacoemulsification combined with intraocular lens (IOL) implantation, the standard cataract surgery internationally, has the advantages of safe, effective, time-saving, and short recovery time, but the lens capsule opacification happening after operation has a severe impact on the comeback of patients' visual function. The current studies believe that this common complication is due to the proliferation, migration, epithelial-mesenchymal transition (EMT), matrix deposition, and contraction taking place in residual lens epithelial cells (LECs) [2]. Scholars found that LECs include two types, type A and type E LECs. Type A LECs that are located under the anterior capsule have two major functions, EMT and migration towards the equatorial and posterior capsule, whereas equatorial type E LECs with a superior proliferation ability play a critical role in the formation of capsular pearls [3]. In the process of routine operation, most of type A LECs can be eliminated via capsulorhexis, while both type A LECs around the anterior capsule and whole type E LECs hidden by the iris are difficult to remove.

Lens capsule opacification can be divided into anterior capsule opacification (ACO) and posterior capsule opacification (PCO) [4] according to the position. ACO, which has the prevalence of 0.47%∼3.3% in the adult [5], mainly shows a white fibrous opacification around the anterior capsular opening and often results in capsule contraction syndrome manifested by the contraction of the anterior capsular opening and the capsular bag, IOL decentration or tilt [6], refractive change, and decreased vision. PCO is mainly caused by the proliferation and EMT of migrated LECs on the originally acellular posterior capsule even the surface of IOL [7]. PCO, with the adult prevalence of 0.3%∼28.4%, usually leads to astigmatism, monocular diplopia, and a decline of visual or contrast sensitivity, and so on. Nearly all children that received cataract surgery had lens capsule opacification [8] because the younger individuals have a greater number of LECs and more suitable levels of hormones as well as cytokines perfect for LECs' growth.

There is not an optimal treatment measure for lens capsule opacification, and an acknowledged method for treating ACO does not appear so far. The common method for treating PCO is Nd : YAG laser capsulotomy. It is effective and helpful to recover the vision and resolve the contraction, but this treatment may cause transient inflammation of the iris or vitreous body triggered by the free capsular debris and lead to macular cystoid edema or retinal detachment [5]. Therefore, how to prevent lens capsule opacification by inhibiting, clearing, or killing LECs becomes a hot area of research. It is generally acknowledged that this common surgical complication is mainly caused by LECs' proliferation, migration, and EMT, and studies have been finding substantial drugs that are useful for controlling LECs. In this review, we summarized these drugs and elaborated the potential mechanisms under their efficacy as thorough as possible.

## 2. Inhibiting the Proliferation of LECs

LECs' proliferation, which can be stimulated by cataract surgery injury, is one of the key pathological processes in lens capsule opacification. Drugs that can hinder cell proliferation cover anti-inflammatory medicine, antineoplastic agents, and immunosuppressive agents, so scholars explored if these drugs can inhibit LECs' proliferation likewise.

### 2.1. Anti-Inflammatory Drugs on LECs' Proliferation

Anti-inflammatory drugs contain hormones and nonsteroidal anti-inflammatory drugs (NSAIDs). Hormones have a rapid and strong anti-inflammatory effects, such as tobramycin dexamethasone eyedrop that can treat a wide scope of ocular infections, but hormones are unsuitable for the prevention of lens capsule opacification as they themselves have a risk of generating cataract. NSAIDs, another kind of anti-inflammatory drugs, exert the anti-inflammatory function by inhibiting cyclooxygenase (COX). COX has two types of isoenzymes, one is COX-1 that has no correlation with inflammation and can induce peptic ulcer if inhibited; the other is COX-2 that is produced under the inducement of injury or inflammatory cytokines and forms the basis of NSAIDs' anti-inflammation action. COX-2 can be specifically hindered by selective NSAIDs such as celecoxib, and previous studies found the elevated level of COX-2 after cataract occurred. Davis et al. [9] found that 20 *μ*mol/L celecoxib could certainly inhibit canine LECs' proliferation in vitro by observing the cell confluence times, but the optimal administration time was 4 days, which was inappropriate in real cataract operation. But this study attempted to implant acrylic IOLs loaded with celecoxib into canine eyes in vitro, which provided us with an administration method worthy of reference. This way not only avoids adding additional operative time, but also ensures the effective dosage by giving drugs full access to the lens capsule.

### 2.2. Antineoplastic Agents on LECs' Proliferation

There are generally two kinds of antineoplastic agents, cytotoxic drugs and noncytotoxic drugs, and several studies showed that both of them can impede LECs' proliferation. Cytotoxic drugs, such as fluorouracil (5-FU) and methotrexate (MTX), have the function of inhibiting tumor cells' proliferation directly with the mechanism of disturbing tumor cells' nucleic acids and proteins. Clinically, 5-FU is widely applied to cure digestive system neoplasms, but often damages the gastrointestinal tract, bone marrow, and even the eyes. In order to decrease the toxicity of 5-FU, Huang et al. [10] tried to inject 5-FU carried by chitosan nanoparticle into the lens capsule after the removal of lens cortex when performed rabbit cataract surgery in vivo and compared this new method with injecting 5-FU directly via cell counting kit-8 (CCK-8). The result proved that 5-FU could curb LECs' proliferation to a certain extent, and the inhibitory concentration 50 (IC_50_) of 5-FU carried by chitosan nanoparticle was only one-fifth of direct administration, which significantly lowered its damage to the cornea, iris, and ciliary body. It was a valuable method that can give consideration to effectivity and safety simultaneously, but the sample size was so small that must be verified by further studies. In addition, former research has found that other cytotoxic drugs, such as daunorubicin and mitomycin C, could also inhibit LECs' proliferation, and similarly, they need suitable use method to reduce the toxicity to intraocular tissues.

Noncytotoxic drugs, such as epithelial growth factor (EGF) receptor inhibitors like gefitinib and erlotinib, monoclonal antibodies, and glucocorticoids, can target the key element of tumor pathogenesis. Wertheimer et al. [6] processed human LECs in vitro by different concentrations of gefitinib and found that gefitinib could hinder LECs' proliferation with the IC_50_ range of 5 *μ*mol/L∼25 *μ*mol/L through the tetrazolium dye-reduction assay (MTT), but the defect was the absence of live animal experiment. In fact, although gefitinib is obviously targeted and effective for non-small-cell lung carcinoma (NSCLC), yet it often causes severe damage to the skin, gastrointestinal tract, and even the eyes. At present, gefitinib is usually restricted to second-line regimens for patients with terminal or severely metastasized tumors, so further research that can prove the feasibility and safety of gefitinib is still in need.

### 2.3. Immunosuppressive Agents on LECs' Proliferation

The immunosuppressive agents, including sirolimus, cyclosporine A (CsA), and tacrolimus, belong to a category of drugs that can inhibit body's abnormal immunity as well as alleviate tissue injuries and are often used for curbing graft rejection after organ transplantation such as corneal transplantation [11]. Previous studies confirmed that both sirolimus and CsA could hinder LECs' proliferation in vitro with the dose–response relationship. To prove the conclusion sufficiently, Liu et al. [12] applied three administration methods of sirolimus to rabbit cataract operation model in vivo, which included 5 ng/mL sirolimus irrigation solution intraoperatively, 2 mg/mL sirolimus eye drop given after surgery, or IOL that contains sirolimus loaded by poly lactic-co-glycolic acid (PLGA). They found that the way of using sirolimus-loaded IOL had the best efficacy in inhibiting LECs' proliferation as well as the onset of capsule opacification, via the methods of slit lamp microscopy, histological observation, and proliferating cell nuclear antigen (PCNA) examination.

In 2016, Teng et al. [13] tried for implanting IOL coated with CsA loaded by PLGA into rabbit eyes and found that this way could inhibit LECs' proliferation as well as lower the PCO grading in 6 months after operation. Moreover, two studies for sirolimus and CsA above found that groups that implanted drug-coated IOL had relatively slighter anterior chamber responses than the control, but the toxicity of the two drugs needs to be detected. Attention should be directed to the superiority of delayed-action drug delivery systems such as chitosan nanoparticle and PLGA, which can reduce drug dosage, add action time, and lower adverse action. Moreover, recent studies suggested that Ozurdex [14], the trade name for the combination of dexamethasone and PLGA, showed good curative effect on diabetic macular edema via implantation intraocularly, which proved a fine biocompatibility of PLGA. These discoveries may offer us the new idea of innovative dosage forms for preventing lens capsule opacification effectively.

### 2.4. Signaling Pathways on LECs' Proliferation

Biochemical reactions of cells are based on a series of different proteins, and each chain of proteins named a signaling pathway. Several signaling pathways, including phosphoinositide 3-kinase (PI3K)/Akt pathway [15], Rho kinase pathway [16], Smad pathway [17], and mitogen-activated protein kinase (MAPK) pathway [18], have been pointed out to involve the pathogenesis of lens capsule opacification. Kayastha et al. [15] found that both andrographolide and LY294002, a known PI3K inhibitor, could decrease the level of activated Akt kinase by Western blotting and explored that the inhibitory effect of andrographolide on human LECs' proliferation in vitro exhibited dose–response relationship with the IC_50_ of 1 *μ*mol/L via BrdU incorporation assay. The results explained that andrographolide could hinder LECs' proliferation via interfering the PI3K/Akt signaling pathway, but this function still needs to be proved by experiment in vivo.

DNA methyltransferase inhibitor, a kind of novel targeted agent for treating polycythemia vera, has passed the phase I clinical trial. Zhou et al. [18] explored whether Zebularine, a DNA methyltransferase inhibitor, could inhibit the proliferation of human LECs in vitro. The results showed that the inhibitory effect exhibited dose–response relationship, and they found that Zebularine had the ability of decreasing the level of Akt as well as MAPK proteins via Western blotting. Those results explained that the mechanism that Zebularine inhibited LECs' proliferation might include PI3K/Akt and MAPK signaling pathways, but the drug safety is still unknown as this study did not explore if Zebularine would influence other intraocular tissues. Furthermore, interactivity between signaling pathways, like Akt being a critical collection point of signaling pathways associated with different biological activities, shows that the relationship between drugs that influence signaling pathways and LECs' proliferation is complicated and needs to be elaborated thoroughly.

## 3. Hindering the Migration of LECs

### 3.1. Antineoplastic Agents on LECs' Migration

MTX, another cytotoxic antineoplastic agent, can treat cancer via effectively inhibiting the tetrahydrofolate dehydrogenase that is indispensable to the synthesis of DNA and is often used for treating ovarian neoplasms, breast neoplasms, and lymphoma that includes intraocular lymphoma. Kassumeh et al. [19] implanted hydrophobic or hydrophilic acrylic IOLs, which [18] were sprayed with PLGA-loaded MTX, into the capsular bag models from human donors. The results of cell scratch assay showed that MTX had the function of hindering LECs' migration, and the better effect was observed in the hydrophobic IOLs group. However, this research found that MTX could lower the viability of corneal endothelial cells and MTX often caused serious bone marrow suppression in practical application; hence how to reduce the toxicity of MTX is the importance of research next.

Except regulating cell proliferation, EGF can also administrate cell migration, extracellular matrix cross-linking, and cell adhesion. Wertheimer et al. [20] explored the effect of erlotinib, another EGF receptor inhibitor, on human LECs, corneal endothelial cells, and retinal pigment epithelium cells in vitro. The research found that erlotinib of 5 *μ*mol/L, 10 *μ*mol/L, and 25 *μ*mol/L could inhibit LECs' migration obviously via Boyden's chamber assay, but erlotinib also caused the viability reduction of corneal endothelial cells and retinal pigment epithelium cells. Previous studies ever reported the cases of corneal ulcer and perforation caused by erlotinib, and erlotinib often triggered the bad adverse reactions of exanthema as well as diarrhea. Hence, both gefitinib and erlotinib are mainly used for metastatic or advanced NSCLC now, and the use of the two drugs for preventing lens capsule opacification needs innovation of ways to decrease toxicity.

### 3.2. Signaling Pathways on LECs' Migration

Rho kinase pathway is related to the occurrence of lens capsule opacification, and its activation excessively can make cells metastasize unlimitedly, which often happens in tumor cells. Lin et al. [16] found that the inhibitory effect of Y27632, a Rho kinase inhibitor, on human LECs' migration in vitro showed dose–response relationship via wound healing assay. Then they implanted IOLs modified by the combination of Y27632 and PLGA into rabbit eyes in vivo, and they observed that the drug could inhibit the occurrence of lens capsule opacification and capsule shrink by slit lamp microscopy. A previous study [21] has shown that Y27362 could not only alleviate inflammatory response of pig corneal endothelial cells, but also inhibit the proliferation and inflammatory cytokine production of human T cell, which might help to explain the potential mechanism of Y27632 that could prevent the occurrence of PCO. However, several studies reported that Y27632 promoted the migration and proliferation of corneal endothelial cells [22], which might remind us that Y27632 has different mechanisms for different cell types, and the specific mechanisms of Rho kinase inhibitors on LECs need further elaboration.

Earlier studies found that alkylphospholipids, a potential kind of antineoplastic agent, could inhibit tumor cells by downregulating the PI3K/Akt signaling pathway. Liegl et al. [23] observed that alkylphosphocholine, one of alkylphospholipids, could inhibit human LECs' migration in vitro via time lapse microscopy, and the results of enzyme-linked immunosorbent assay (ELISA) showed that the inhibitory effect of alkylphosphocholine on PI3K kinase activity exhibited dose–response relationship, which explained that alkylphosphocholine might attenuate LECs' migration via inhibiting PI3k/Akt signaling pathway. The research showed that blocking PI3k/Akt signaling pathway could impede not only LECs' proliferation but also migration. But this research was an experiment in vitro and did not identify the critical pharmacokinetic parameters such as the most suitable drug concentration. In short, both drugs above need to be explored further.

### 3.3. Genetic Therapy on LECs' Migration

Genetic therapy, a remedy on the level of nucleic acids, can treat single genetic diseases, neoplasms, and metabolic diseases. MicroRNAs, also called miRNAs, are a kind of noncoding RNAs expressed endogenously and usually combined with targeted mRNAs to suppress the expression of targeted genes. Studies showed that miRNAs participated in a wide range of biological processes, such as cell differentiation, growth and development, and oxidative stress resistance [24]. Dong et al. [25] found that the human LECs transfected by miRNA-181a in vitro had a lower migration ability than normal, and the Western blotting result reflected that miRNA-181a could decrease the level of COX-2, a factor that was related to lens capsule opacification. These results showed that miRNA-181a was expected to become a genetic therapy target for lens capsule opacification.

Another potential target for gene therapy of lens capsule opacification after cataract surgery is miRNA-34a, an miRNA that can inhibit the migration of human LECs in vitro via transwell migration assays, and the results of Western blotting showed that it could also reduce the level of activated AKT as well as ERK, the extracellular signal-regulated kinase of MAPK signaling system. This study implemented by Feng et al. [26] showed that miRNA-34a might contribute to the prevention of lens capsule opacification via inhibiting PI3K/AKT signaling pathway and MAPK pathway. However, on the one hand, the gene expression of cataract patients differs from healthy people, which adds the difficulty of finding an exact gene target for the prevention of lens capsule opacification. On the other hand, a previous study by Malecaze et al. [27] found that a gene therapy method, which include injecting the adenovirus vector with transduced ectopic gene into the rabbit capsular bag in vivo, could not ensure that the transgenic expression was strictly limited to the residual LECs after cataract surgery without impacting other ocular cell types such as corneal endothelial cells. In summary, genetic therapy is generally situated in the research phase, and it is imperative to find highly specific gene target for residual LECs in the future.

## 4. Impeding the EMT of LECs

Cell transdifferentiation is composed of two forms, one is the cell plasticity that is mainly related to neurons; the other is EMT that includes a series of pathophysiological events in epithelial cells. EMT usually manifests as the loss of cell polarity and adhesion ability, acquisition of the migration and invasiveness power, production of substantial extracellular matrix, and the impediment of apoptosis. The EMT process is often found in the processes of lens capsule opacification, wound healing, and neoplasm metastasis.

### 4.1. Signaling Pathways on LECs' EMT

In 1982, Greenburg et al. [28] put the LECs separated from lens capsule into the culture media that contains collagen gels and observed that the LECs transformed from cuboidal cells to spindle myofibroblasts, which symbolized the occurrence of EMT. In addition to the change of cell shapes, the common EMT markers include *α*-smooth muscle actin (*α*-SMA), fibronectins, and vimentin, and the reduction of these markers showed that EMT was inhibited. Zukin et al. [29] selected the aldose reductase (AR) transgenic mice as the AR overexpression model and found that AR overexpression could increase the level of *α*-SMA and fibronectins in mice LECs via real-time Polymerase Chain Reaction (PCR), which reflected that AR overexpression activated LECs' EMT. After adding AR inhibitor Sorbinil, they observed the decline in the level of *α*-SMA and fibronectins, which meant the EMT of LECs was inhibited. This discovery was verified by Chang et al. [30] again, and furthermore, they found that Sorbinil could also reduce the level of activated ERK that belongs to MAPK signaling system. Both studies showed that AR inhibitor Sorbinil might contribute to preventing capsule opacification via blocking MAPK signaling pathway, which could inhibit LECs' proliferation and EMT simultaneously. However, a novel study [31] found that Sorbinil has the ability of promoting lens regeneration, another process that happened with EMT of LECs after cataract surgery, which may weaken the possible function of AR inhibitor Sorbinil in preventing lens capsule opacification.

Smad signaling pathway is a classic pathway for EMT, and transforming growth factor (TGF)-*β*, its upstream signaling molecule, can activate this pathway by inducing the phosphorylation of Smad protein. TGF-*β* plays a key role in LECs' EMT [32] and is often used for building LECs' EMT model. Ning et al. [17] discovered that THZ1, a potential antineoplastic agent, could inhibit not only cyclin-dependent kinases of tumor cells, but also the EMT of LECs activated by TGF-*β*. The result of Western blotting showed that THZ1 lowered the level of active Smad, *α*-SMA, and fibronectins, which meant the possible mechanism of THZ1 in impeding LECs' EMT is the inhibition of Smad signaling pathway. Similarly, Raghavan et al. [33] detected that an endogenous ligand for a receptor for advanced glycation end products (EN-RAGE) could also reduce the level of *α*-SMA, active Smad, and TGF-*β* of human LECs in vitro with the results of quantitative real-time PCR, which showed that EN-RAGE might also attenuate the EMT of LECs via inhibiting Smad signaling pathway. Drugs above are still in the stage of examination, and their definite pharmacologic actions and mechanisms need to be elaborated in the next step.

### 4.2. Genetic Therapy on LECs' EMT

As previously mentioned, miRNAs have a wide range of biological functions. Han et al. [34] compared the miRNA-34a level in LECs of cataract patients with normal human donor eyes and found that cataract patients had a lower miRNA-34a level than normal. After transfecting TGF-*β* induced LECs' EMT models with miRNA-34a, the result of Western blotting showed that the EMT markers, *α*-SMA, fibronectins, and vimentin, decreased and the epithelial cells' marker E-cadherins increased. The discovery with a study for miRNA-34a above verified that miRNA-34a could hinder LECs' migration as well as EMT, which might provide a relatively reliable gene therapy target for lens capsule opacification. Moreover, this study detected that the transcription factor Notch1 elevated in LECs transfected with miRNA-34a via quantitative real-time PCR, which may provide a new therapeutic target.

In addition, both miRNA-497-5p [35] and miRNA-30a [36] were found to have the ability of inhibiting the EMT of human LECs induced by TGF-*β* in vitro, and the results of Western blotting showed that these two miRNAs could reduce the level of vimentin and *α*-SMA, EMT markers, and increase the level of E-cadherins, an epithelial cells' marker, which verified their function of inhibiting EMT of LECs. And miRNA-497-5p could downregulate the level of activated Smad, which might remind us that the potential inhibition mechanism of miRNA-497-5p on EMT of LECs included the inhibition of Smad signaling pathway. Wang et al. [35] found that the function mechanism of TGF-*β*, which referred primarily to the more possible occurrence of lens capsule opacification by promoting LECs' EMT, contained dozens of miRNAs, and miRNAs associated with lens capsule opacification have been under study, which may help to identify the therapy targets for lens capsule opacification.

### 4.3. Other Medicines on LECs' EMT

The EMT of left LECs after cataract surgery is closely related to the lens capsule opacification and capsular fibrosis. Resveratrol, one of the natural polyphenols, has the function of inhibiting fibrosis and antioxidants. Smith et al. [37] added 30 *μ*mol/L resveratrol into human LECs in vitro, and the results of quantitative real-time PCR showed that resveratrol could decrease the level of not only EMT markers, *α*-SMA and fibronectins, but also TGF-*β* activator, matrix metalloproteinase (MMP) 2. This study explained that resveratrol was more likely to prevent lens capsule opacification via hindering LECs' EMT, but they did not observe and evaluate the effect of resveratrol on other intraocular tissue cells. While Resveratrol has been authorized to the market, it is usually used as a nutrient supplement to improve bone density and protect the cardiovascular system, and further research that elaborates its pharmacologic mechanism is still in need.

Aspirin, one of the common antipyretic-analgesic and anti-inflammatory drugs as well as anticoagulants, has several functions of analgesia, treating inflammation, preventing thrombosis after operation, and guarding against the onset of myocardial infarction. Nam et al. [38] detected that aspirin could reduce the level of *α*-SMA as well as fibronectins and increase histone H3, but had no effect on Smad and ERK protein of human LECs in vitro. These results showed how aspirin inhibiting LECs' EMT was relatively complicated and still needed further study. Vitamin C, one of indispensable vitamins, has wide and robust pharmacologic actions such as enhancing body immunity. The latest research showed that oral vitamin C could reduce astigmatism and relieve pain, foreign body sensation, and photophobia after cataract surgery [39], and vitamin C injection could treat chronic iron poisoning and methemoglobinemia. Zhao et al. [40] discovered that human LECs' EMT could be impeded by 100 *μ*mol/L vitamin C in vitro and that the activity of hypoxia-inducible factor (HIF)-1*α* being blocked might explain the possible action mechanism. However, experimental subjects of two studies were human LECs in vitro; the conclusions and effects should be verified in vivo next.

## 5. Clearing or Killing LECs

### 5.1. Ways of Clearing LECs

In physical state, LECs adhered to the capsule and there were junctional complexes that included desmosomes between LECs, with the extracellular matrix playing a key role in the conjunction among LECs as well as between LECs and the capsule. The critical materials covered hyaluronic acids, collagenous fibres, and glycoproteins. If junctions among LECs as well as between LECs and the capsule are broken, cooperating with rinse during operations, the goal of clearing LECs can be achieved.

#### 5.1.1. Cell Dissociation Solutions on Clearing LECs

Cell culture technology usually applied cell dissociation enzymes to isolate cells from diversified tissues, and trypsin, dispase, fibrinolysin, and hyaluronidase were ever used to remove LECs from the capsule to help eliminate LECs. Trypsin and dispase had a relatively strong function of dissociating LECs and could destroy cellular structures even if mixed with sodium hyaluronate, so they are only applied in LECs culture in vitro. Fibrinolysin and hyaluronidase were used to induce posterior vitreous detachment, and fibrinolysin could separate LECs from the lens capsule by dissolving fibres. But the lens capsule is a fibre-enriched and stretchy basement membrane, which can also be destroyed by fibrinolysin; hence, fibrinolysin is seldom used actually. Hyaluronidase contributed to clearing LECs via dissolving hyaluronic acid among LECs and weakening extracellular matrix viscosity. However, it was reported that hyaluronidase could aggravate inflammatory responses after cataract surgery [41], limiting its wide application clinically.

Edetic acid (EDTA), a chemical chelating agent, can weaken cell adhesion by binding to calcium and magnesium ions, and research in vitro proved that EDTA could detach LECs from the capsule. Hazra et al. [42] injected EDTA into the rabbit capsule after removing lens cortex and discovered that EDTA could reduce the occurrence of lens capsule opacification after surgery. Moreover, the results of intraocular pressure, corneal endothelial cell density, and electroretinography after surgery showed no noticeable difference between EDTA group and the control, which explained the relative safety of EDTA. The zymography result exhibited the reduction of MMP2 activities, which hinted that EDTA could not only contribute to clear LECs, but also inhibit LECs' EMT, and it is desirable to do clinical trials about EDTA.

#### 5.1.2. Clinical Drugs on Clearing LECs

The white lens opacity in many cataract patients made the lens anterior capsule difficult to see; sometimes coloring agents were needed to make the anterior capsule visible and easy to do capsulorhexis. Trypan blue, indocyanine green (ICG), and brilliant blue, common coloring agents, were ever applied in surgery for macular diseases to assist with internal limiting membrane peeling. Melendez et al. [43] found that ICG above 0.1 mg/mL had toxicity to human LECs in vitro, but 5.0 mg/mL trypan blue did not affect LECs' viability obviously. Later, Sharma et al. [44] attempted to inject 0.2 mL of 0.1% trypan blue into the lens capsule during hydrodissection in human cataract surgery and discovered that the PCO score in the group with trypan blue injection was lower than the control in the 12 months after operation, which showed that trypan blue was beneficial to prevent lens capsule opacification by clearing LECs during surgery. A recent research found that trypan blue could attenuate capsule elasticity [45], so whether coloring agents are suitable for guarding against lens capsule opacification still needs further research.

Lidocaine, a local anesthetic agent, and balanced salt solution (BSS) that belongs to washing fluid are usually used in cataract surgery. Vargas et al. [46] injected 1% lidocaine or BSS into rabbit lens capsule in vitro, supplemented by rinsing the capsule, and found that using lidocaine for 2 minutes could detach LECs from the capsule while BSS could not. But lidocaine led to LECs' death partially, and it is necessary to evaluate the effect of lidocaine on intraocular tissue cells like corneal endothelial cells later. Hypertonic saline solution has a high osmotic pressure that can cause cell water loss and shrinking, but surrounding tissues will be ruined by the excessive osmotic pressure. Zhang et al. [47] found that 400 mOsm/L hypertonic saline solution coordinated with BSS rinse could effectively clear human LECs in vitro, but must add Na-K-Cl transporter to prevent the recovery of cell volume by regulation. The method was relatively complicated, and this research did not involve the observation about the effect of hypertonic saline solution on the capsule mechanical characteristics and surrounding intraocular tissues.

Distilled water (DW), also called sterile water for injection, is usually used as the solvents of sterilized powder for injection and thinners for injection and is a washing fluid widely used in surgery. Its osmotic pressure is approximately 0, which is obviously lower than the osmotic pressure of human tissue cells that is within 280 mmol/L∼310 mmol/L, and DW can cause cells bursting by absorbing water to help eliminate cells. Scholars [3, 48] infused the capsule with DW for 3 minutes after removing the lens cortex in human cataract surgery and proved that DW could reliably eliminate LECs and decrease the occurrence of lens capsule opacification. For protecting intraocular tissues, Rekas et al. [3] used a sealed-capsule irrigation (SCI), a specific device that could close the capsule and prevent drugs extravasation, but SCI had a fixed size that was not suitable for some patients and patients who are prepared to have microincisional cataract surgery. Zhang et al. [48] made use of air to sustain the anterior chamber and protect intraocular tissues with the vitrectomy equipment and special syringe made independently, which was effective but added difficulty and complication of surgery. Hence, how to take advantage of DW to prevent lens capsule opacification safely and efficaciously still needs innovation of surgical methods.

### 5.2. Methods of Killing LECs

Cell death covers autophagy, apoptosis, and necrosis, with the definite manifestations of cell lysis, cell swelling, cell vacuolization, organelles destruction, karyorrhexis, pyknosis, and so on. Drugs that can lead to the above manifestations in LECs, such as some immunosuppressive agents, hydrogen peroxide and sanguinarine, are more likely to be used for preventing lens capsule opacification clinically by inducing LECs' death. Chandler et al. [49] observed that the canine LECs treated with CsA produced substantial autophagosomes as well as vacuole formation in vitro and had a higher level of LC3-II, one of the autophagy-related proteins, by Western blotting. After adding autophagy inhibitor 3-methyladenine (3-MA), the effect of CsA would be controlled accordingly, which reminds us that CsA could kill LECs by inducing LECs autophagy, and its function as well as safety must be verified via animal experiments in vivo.

Tian et al. [50] observed the killing effect of sirolimus on human LECs in vitro via terminal deoxynucleotidyl transferase nick end labeling (TUNEL) detection, and the result of Western blotting showed that sirolimus could increase the level of apoptosis induction protein Bax and lower the Bcl-2 level, one of the antiapoptotic proteins. Those findings explained that sirolimus might kill LECs via inducing apoptosis. Li et al. [51] explored that the increase of LECs apoptosis could be caused by miRNA-15a transfection or 200 *μ*mol/L hydrogen peroxide via flow cytometry. Results of Western blotting showed that both miRNA-15a and hydrogen peroxide could reduce the level of Bcl-2 protein and E2F3 transcription factor, one of the cell cycle proteins, which showed that the killing effect of miRNA-15a and hydrogen peroxide on LECs was via inducing LECs apoptosis probably. However, several researchers thought that LECs' apoptosis belonged to the main pathogenesis of cataract [52], and the relationship between LECs' apoptosis and the prevention of lens capsule opacification needs further exploration.

## 6. Conclusions

LECs, a simple cuboidal epithelium, are located at the anterior and equatorial lens capsule physiologically, and their biological activities are in dynamic balance. But the trauma of cataract surgery can break this balance, causing the destruction of blood-aqueous barrier (BAB) and the release of several cytokines such as TGF-*β*, interleukins, and interferons. These reactions can induce LECs' proliferation, migration, and EMT, which will lead to lens capsule opacification eventually and affect the visual function again. Long-term, substantial studies found that several drugs could reduce the occurrence of lens capsule opacification via inhibiting, clearing, or killing LECs, and sometimes, a same drug could act on LECs via multiple mechanisms. For instance, sirolimus and CsA, two immunosuppressive agents, could not only inhibit LECs' proliferation [12, 13], but also kill LECs [49, 50]. The administration mode, a key factor that influences the pharmacological action, includes eyedrop [12], injection during hydrodissection [44] or after removing the lens cortex [48], and implantation of IOL combined with drugs [9]. Researchers thought that the method of injecting drugs into the capsule during cataract surgery could not only make drugs have full access to LECs, but also satisfy the clinical need of simplicity, quickness, and practicability. And it is also suitable for children who are young and difficult to cooperate with surgery but almost have lens capsule opacification after cataract surgery in all of them.

Moreover, the pathogenesis of lens capsule opacification is relatively complicated, which contains various biological behaviors, including LECs' proliferation, migration, and EMT, many signaling pathways, and a number of key molecules. Although a number of drugs could reduce the appearance of lens capsule opacification via interfering LECs, there is not a drug that can effectively inhibit LECs and have a low toxicity to intraocular tissues simultaneously. In summary, substantial efforts about elaborating the specific pathogenesis of lens capsule opacification, the successful establishment of human and animal LECs cell lines in vitro, and the normative and widely implemented animal cataract surgery models lay the foundation for finding an optimal and suitable drug to prevent lens capsule opacification, and it is more likely for us to tackle this common complication that affects the visual function severely in the near future.
